# Additional Recommendations on Assessment of Left Ventricle End‐Diastolic Volume

**DOI:** 10.1002/clc.70029

**Published:** 2024-10-21

**Authors:** Fatma Nur Toksöz, Özden Seçkin Göbüt, Serkan Ünlü

**Affiliations:** ^1^ Cardiology Department Gazi University Ankara Turkey

Dear Editor,

We read your article, “Revisiting Echocardiographic Ranges of Left Ventricular End Diastolic Volume Index,” with great interest [1]. As numerous studies demonstrate, the end‐diastolic diameter is not an adequate substitute for left ventricular end‐diastolic volume (LVEDV) in evaluating dilated left hearts. We agree with your emphasis on LVEDV as a critical diagnostic parameter. However, we offer some observations and suggestions to enhance the discussion further.

The reclassification of the LVEDV index (LVEDVi), particularly among women, is significant. The fact that nearly 20% of female subjects were reclassified into higher abnormal categories underscores the need for gender‐specific cutoffs in future guidelines. The 2015 guidelines disproportionately affect women, making further research into gender‐specific physiological differences vital for refining thresholds. Addressing these distinctions would better align future guidelines with the diagnostic needs of female patients.

We appreciate your acknowledgment of regional variability in left ventricle (LV) parameters, supporting the need for international, multicenter studies. This approach would help establish reference ranges that are more globally representative, especially since your study noted differences in LV size parameters across countries. A global collaborative effort would account for body composition and cardiac anatomy variability worldwide.

While the 2015 guideline introduced useful refinements, we agree that clinical outcomes should play a greater role in determining classifications. Misclassification could lead to unnecessary diagnostic testing, increasing healthcare costs and patient anxiety. Shifting toward outcome‐based classification systems may reduce overdiagnosis and better target interventions for those at real risk.

Your study demonstrates that significant aortic and mitral regurgitation can impact LVEDVi classification [1]. Further exploration of how controlling for these and other comorbidities could clarify the extent to which LVEDVi changes are due to actual left ventricular enlargement. A more homogeneous study population would enhance the precision of conclusions.

Lastly, the limitations of using body surface area as an indexing method, particularly for individuals with extreme body compositions, are important [2, 3]. We agree that alternative methods, such as length‐based scaling, could offer a more accurate reflection of LV size in obese or extremely thin individuals. Investigating these alternative indexing methods would improve diagnostic accuracy across diverse body types.

Once again, we commend you on this important contribution to the field and look forward to future research that builds on these findings.

Sincerely,

## Conflicts of Interest

The authors declare no conflicts of interest.

## Data Availability

The authors have nothing to report.
